# Ventilator-associated pneumonia caused by *Chryseobacterium indologenes*: a rare infant case and review of the literature

**DOI:** 10.1186/s40064-016-3449-x

**Published:** 2016-10-07

**Authors:** Serkan Atıcı, Zeynep Alp Ünkar, Kübra Erdem, Eda Kepenekli Kadayifci, Ayşe Karaaslan, Aslı Çınar Memişoğlu, Ahmet Soysal, Nurver Ülger Toprak, Güner Söyletir, Eren Özek, Mustafa Bakır

**Affiliations:** 1 Department of Pediatrics and Division of Pediatric Infectious Diseases Medical School, Marmara University, Istanbul, Turkey; 2 Department of Pediatrics and Division of Neonatology, Medical School, Marmara University, Istanbul, Turkey; 3 Department of Microbiology, Medical School, Marmara University, Istanbul, Turkey; 4 T.C. Sağlık Bakanlığı-Marmara Üniversitesi Pendik Eğitim ve Araştırma Hastanesi, Fevzi Çakmak Mah. Mimar Sinan Cad. Üstkaynarca, Pendik, Istanbul, Turkey

**Keywords:** *Chryseobacterium indologenes*, Infant case, Ventilator-associated pneumonia

## Abstract

**Background:**

*Chryseobacterium indologenes* is an uncommon organism that has been documented to cause a variety of invasive infections mostly in hospitalized patients with severe underlying diseases.

**Case presentation:**

A three-month-old female infant born at term by caesarean section with meningomyelocele and congenital diaphragmatic hernia had two surgeries for the repair of meningomyelocele and diaphragmatic hernia on her 3rd and 14th day, respectively. On the 3rd month of her life, she deteriorated clinically with fever, leukocytosis and increase of acute-phase reactants. Gas exchange condition became worse than it was before. Respiratory secretions, oxygen requirements and ventilator demand increased. Chest X-ray showed bilateral pulmonary infiltrates. Bacteriological blood, urine and cerebrospinal fluid culture test results were negative. *C. indologenes* was isolated from tracheobronchial secretion sample obtained by endotracheal aspiration. Although susceptible to ciprofloxacin (MIC:0.5 gr/L), levofloxacin and piperacillin–tazobactam, the isolate was resistant to meropenem, imipenem and colistin. She was treated with ciprofloxacin successfully. Her fever resolved and gas exchange condition improved after 72 h of the treatment. The antibiotic treatment was given for a course of 14 days.

**Conclusion:**

*Chryseobacterium indologenes* may emerge as a potential pathogen in infants with the factors such as invasive equipment, having underlying diseases and prolonged hospitalization.

## Background

*Chryseobacterium indologenes* is a Gram-negative, aerobic, non-fermenting, non-motile, catalase-, oxidase-, and indole positive bacillus. It is widely distributed in environmental sources including water, soil and plants (Omar et al. 2014). It is possible that physicians may encounter this pathogenic microorganism in hospital environment such as mechanical ventilator circuits. *C. indologenes* is a very rare pathogen in human that has been reported to cause infections mostly in hospitalized patient with immunocompromised conditions or infants. *C. indologenes* is inherently resistant to many antimicrobial agents including carbapenems (Omar et al. 2014).

In our case, *C. indologenes* was isolated from a tracheobronchial secretion sample in a 3-month-old infant diagnosed with ventilator-associated pneumonia.

## Case description

**Fig. 1:**
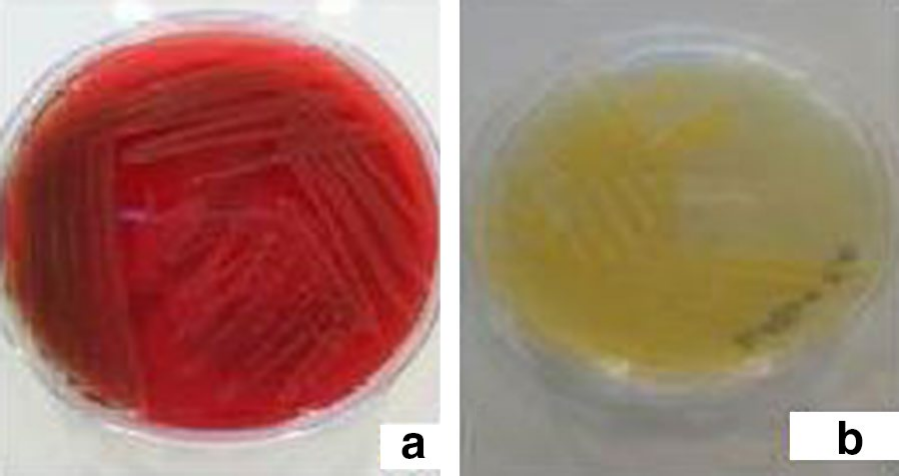
Yellow colonies of *Chryseobacterium indologenes* on sheep blood agar (**a**) and Müller-Hinton agar (**b**)

A three-month-old female infant born at term by caesarean section was prenatally diagnosed with meningomyelocele and congenital diaphragmatic hernia and was transferred to the neonatal intensive care unit (NICU) for further management. Because of severe dyspnea, she was intubated and given mechanical ventilatory support. She had two surgeries for the repair of meningomyelocele and congenital diaphragmatic hernia on the 3rd and 14th days of life, respectively. VP shunt was inserted when she was one month old because of hydrocephalus. The patient had a bacteremia caused by *Stenotrophomonas maltophilia*. The pathogen was susceptible to ceftazidime and ciprofloxacin and treated with ceftazidime. After the completion of treatment period, the patient remained antibiotic-free for 7 days. While she was monitored on mechanical ventilation on the 3rd month of life, she clinically deteriorated with fever (38.5 °C). Gas exchange condition became worse than it was before. Respiratory secretions, oxygen requirements and ventilator demand increased. Her laboratory findings showed leukocytosis with increased number of neutrophils (WBC: 14,500/mm^3^, neutrophils: 8600/mm^3^) and high levels of acute phase reactant (C-reactive protein: 19.2 mg/dl). Chest X-ray showed bilateral pulmonary infiltrates compatible with pneumonia. Blood, cerebrospinal fluid, urine and tracheobronchial secretion specimen obtained by sterile endotracheal aspiration were sent to microbiology laboratory for bacterial culture. Increased leukocytes were observed on smear of tracheobronchial secretion sample. Previous infection history caused by *Stenotrophomonas maltophilia* bacteremia was considered and empiric antibiotic therapy with vancomycin, ceftazidime and ciprofloxacin were started. Tracheobronchial secretion obtained by sterile endotracheal aspiration yielded yellow-colored colonies after 24 h incubation on sheep blood agar (Fig. 1a). Similar yellow-pigmented colonies were also observed on Müller-Hinton Agar (Fig. 1b). *C. indologenes* was identified by conventional methods, VITEK 2 ID-AST (bioM´erieux, France) fully automatized system and Matrix-Assisted Laser Desorption/Ionization time-of-flight, Mass Spectrometry(MALDI-TOF MS). Based on sequencing result of partial 16S rRNA gene, the isolate matched 99 % identities with the region from 852 to 860 bp of the 16S rRNA sequence of *C. indologenes* strain (GenBank sequence ID: LN681561.1). There was no other co-pathogen. Antimicrobial susceptibility testing was performed by both determining the minimal inhibitory concentration (MIC) value using microdilution method and measuring the inhibition zone diameter onto Mueller–Hinton agar (Oxoid Ltd., Basingstoke, UK) medium aerobically at 35 ± 2 °C for 18–24 h using Kirby-Bauer’s disk diffusion method according to Clinical and Laboratory Standards Institute (CLSI) guidelines for non-fermenting microorganisms. Antimicrobial susceptibility testing of the organism revealed resistance to aminoglycosides, ceftazidime, meropenem, imipenem, colistin and was susceptible to ciprofloxacin, levofloxacin, piperacillin–tazobactam and cefepime.

Her fever resolved and gas exchange condition improved after 72 h of treatment. The patient gave a good clinical response with the empiric treatment. For this reason, we did not want to change ciprofloxacin, and the treatment was continued with ciprofloxacin monotherapy. Blood, urine and cerebrospinal fluid culture test results were negative. Repeated endotracheal aspiration specimen culture was also negative after 72 h of antibiotherapy. The antibiotic treatment was given for a course of 14 days.

## Discussion

*Chryseobacterium* genus is a group of Gram-negative, aerobic bacilli that belong to Flavobacteriaceae family. *C. indologenes* is the most common species and was first described by Vandamme et al. in 1994 (Vandamme et al. 1994). However *C. indologenes* is not a part of the human microflora, it can be found in water supplies in the hospital environment. Contamination of the medical devices containing water (intubation tubes, respirators, humidifiers, etc.) in hospital settings may lead to severe infections in hospitalized patients. Both long-term colonization with *C. indologenes* of medical devices and invasive infections have been reported Hsueh et al. (1996). It is known that the production of biofilm and protease activity by *C. indologenes* is an important mechanism involved in its virulence although the exact mechanism of pathogenicity is not well determined Hsueh et al. (1996).

*Chryseobacterium indologenes* infections in children are very rare and usually associated with the presence of invasive medical equipment as in our case. It has been reported to cause a variety of invasive infections such as ventilator-associated pneumonia, bacteremia, catheter-related bloodstream infection, lumboperitoneal shunt infection, pyelonephritis, biliary tract infections, peritonitis, ocular infections, surgical site infection, wound infection, endocarditis, and keratitis (Hsueh et al. 1996; Deng et al. 2015; Bayraktar et al. 2007; Douvoyiannis et al. 2010; Al-Tatari et al. 2007). Besides the use of invasive medical devices, other important risk factors for *C. indologenes* infection are use of broad-spectrum antibiotics, underlying diseases and primary or acquired immunosuppressive conditions. Infections caused by *C. indologenes* are associated with a high mortality rate (Nemli et al. 2015).

*Chryseobacterium indologenes* is a rare pathogen isolated from clinical specimens, and its antimicrobial susceptibility pattern is not well defined. The organism has a limited antimicrobial sensitivity. The choice of an effective antibiotic for the empirical treatment is difficult. *Chryseobacterium* organisms produce class A -lactamase and class B carbapenem-hydrolyzing -lactamase molecules that cause intrinsic carbapenem and cephalosporin resistance. *C. indologenes* is usually resistant to aminoglycosides, other -lactams, chloramphenicol, linezolid, and glycopeptides and is usually susceptible to ciprofloxacin, levofloxacin, trimethoprim-sulfamethoxazole (TMP–SMX), and piperacillin–tazobactam (Nemli et al. 2015; Lin et al. 2010). According to the results of the SENTRY Antimicrobial Surveillance Program, the most active antimicrobials against *C. indologenes* are quinolones (≥95 % susceptibility) and trimethoprim–sulfamethoxazole (95 % susceptibility), followed by piperacillin–tazobactam (90 % susceptibility). Ciprofloxacin, cefepime, ceftazidime, piperacillin, and rifampin showed reasonable activity (85 % susceptibility) (Kirby et al. 2004). Due to the limited data in the pediatric age group, a standard and effective treatment for *C. indologenes* infections is still not clear. Our case was ventilator-associated pneumonia caused by *C. indologenes*, which was successfully treated with ciprofloxacin monotherapy.

*Chryseobacterium indologenes* is a widespread bacterium in the environment, in particular on the wet surfaces of hospitals and water systems. Although there is not any outbreak report in pediatric wards, a distillate water tank was shown to be the source of *C. indologenes* that caused a blood stream infection (Bayraktar et al. 2007). The organism may spread because of limited education of the healthcare personnel and incomplete adherence to infection control measures. The physician should report this rare pathogen to infection control department. If necessary, environmental cultures should be performed to identify the source. Healthcare personnel have to be careful and they should be educated about the implementation of infection control measures, especially hand hygiene compliance. We reported this case to our hospital infection control committee. Environmental cultures such as the respiratory circuit, humidifier, etc. were not performed. Contact isolation precautions were applied to the patient, and healthcare workers were educated and reinforced about infection control measures. Outbreak did not occur.

## Review of the literature about *C. indologenes* infections in pediatric age groups

We searched for information about *C. indologenes* infections in the MEDLINE (PubMed, Ovid) database and could able to suitable 24 pediatric cases. Patients were excluded if they were an adult (>18 years) case from this review. The most important characteristics of cases were presented in Table 1. Gender was reported for 23 patients, 12 (52 %) of them were female, and 16 (66.6 %) patients were ≤1 year of age. Most of the patients (n = 21, 87.5 %) had underlying conditions and only 6 (25 %) patients had no medical device. Five patients (1, 18, 20, 21, 23rd patients in Table 1) had co-infections, including *Escherichia coli*, *Morganella morganii*, *Acinetobacter baumannii*, *vancomycin resistant enterococcus*, *Stenotrophomonas maltophilia* and *Burkholderia cepacia*. The most commonly used antibiotics were ciprofloxacin and TMP-SMX. Four patients died and the mortality rate was found 16.6 % in this series.

**Table 1:** Characteristics of pediatric cases caused by *Chryseobacterium indologenes*

No	Age/sex	Underlying condition	Medical device	Infection type	Treatment	Outcome	Year/reference
1	1 year/M	Burn	Ventilator	VAP	Ciprofloxacin, cefoxitin, amikacin	Died	1996/Hsueh et al. (1996)
2	5 year/F	Neuroblastoma	CVC	Bacteremia	NR	Survived	1996/Hsueh et al. (1996)
3	1 year/F	Hepatoblastoma	CVC	Bacteremia	NR	Survived	1996/Hsueh et al. (1996)
4	2 year/M	Diabetes mellitus (Type 1)	Peripheral catheter	Bacteremia	Ceftriaxone	Survived	2005/(Cascio et al. 2005)
5	5 month/M	Down syndrome, diaphragmatic hernia, ASD	Ventilator	Bacteremia	Vancomycin, ofloxacin	Died	2007/(Bayraktar et al. 2007)
6	13 year/M	Congenital hydrocephalus	Lumboperitoneal shunt	Lumboperitoneal shunt infection	TMP–SMX, Rifampin	Survived	2007/(Al-Tatari et al. 2007)
7	33 day/F	None	None	Bacteremia	Cefepime	Survived	2010/(Douvoyiannis et al. 2010)
8	2 month/M	Hydrocephaly	External shunt	Meningitis, sepsis	Ampicillin–sulbactam, levofloxacin	Died	2011/(Ceylan et al. 2011)
9	36 week newborn/NR	Prematurity	Ventilator	Bacteremia	Cefoperazone–sulbactam	Survived	2011/(Sudharani and Asiya Saxena 2011)
10	20 day/M	Complex congenital heart disease	Ventilator	VAP	Piperacillin–tazobactam	Survived	2011/(Calderón et al. 2011)
11	8 day/F	None	None	Meningitis	Cefepime	Survived	2013/(Hendaus and Zahraldin 2013)
12	3 year/F	Acute myeloid leukemia	CVC	CRBSI	Ciprofloxacin, minocycline	Survived	2013/(Kodama et al. 2013)
13	6 month/M	Congenital hydrocephalus, prematurity	Ventriculoperitoneal shunt	Meningitis	TMP–SMX, cefoperazone–sulbactam	Survived	2013/(Ozcan et al. 2013)
14	11 month/M	Holoprosencephaly, obstructive hydrocephalus	Ventriculoperitoneal shunt	Meningitis	TMP–SMX, ceftazidime	Survived	2014/(Olbrich et al. 2014)
15	6 day/F	SGA	None	Meningitis, sepsis	Ciprofloxacin, TMP–SMX	Survived	2014/(Eshwara et al. 2014)
16	3 month/F	ASD	CVC	Bacteremia	TMP–SMX	Survived	2014/(Aydin et al. 2014)
17	27 week newborn/F	Complex congenital heart disease	Central catheter, arterial and venous line	Bacteremia	Ciprofloxacin, imipenem	Survived	2014/(Alford and Shelton 2014)
18	3 month/M	Metabolic disease	CVC, ventilator	CRBSI	Ciprofloxacin, imipenem, colimycin, linezolid	Died	2016/(Aykac et al. 2016)
19	2 year/F	Congenital hydrocephalus	External shunt	Meningitis	Ciprofloxacin, TMP–SMX	Survived	2016/(Aykac et al. 2016)
20	8 year/M	Cystic fibrosis, nephrotic syndrome	None	Pneumonia	Ceftriaxone	Survived	2016/(Aykac et al. 2016)
21	8 month/M	Ileus	CVC	Bacteremia	Ciprofloxacin, meropenem, vancomycin	Survived	2016/(Aykac et al. 2016)
22	16 month/F	ITP, immunosuppressive therapy	None	Bacteremia	Ceftriaxone	Survived	2016/(Aykac et al. 2016)
23	3 year/F	Cerebral palsy	CVC	CRBSI	Meropenem, amikacin	Survived	2016/(Aykac et al. 2016)
24	11 year/F	None	None	Soft tissue infection	Ceftazidime, metronidazole	Survived	2016/(Srinivasan et al. 2016)
25	3 month/F	Meningomyelocele, congenital diaphragmatic hernia	Ventilator	VAP	Ciprofloxacin	Survived	Our case

*M* male, *F* female, *NR* not reported, *TMP*–*SMX* Trimethoprim–Sulfamethoxazole, *VAP* ventilator-associated pneumonia, *CVC* central venous catheter, *CRBSI* catheter-related blood stream infection, *SGA* small for gestational age, *ASD* atrial septal defect, *ITP* immune thrombocytopenic purpura

## Conclusion

*Chryseobacterium indologenes* may emerge as a potential pathogen in infants with risk factors such as invasive medical equipment, underlying diseases, broad-spectrum antibiotics usage and prolonged hospitalization. Physicians should consider this pathogen in the etiology of medical device-associated infections. *C. indologenes* may have resistance to empirically administered antimicrobial treatment for nosocomial infections and antimicrobial susceptibility test results are important to guide the antibiotic treatment.
